# Posttraumatic growth inventory: challenges with its validation among French cancer patients

**DOI:** 10.1186/s12874-022-01722-6

**Published:** 2022-09-24

**Authors:** Yseulys Dubuy, Véronique Sébille, Marianne Bourdon, Jean-Benoit Hardouin, Myriam Blanchin

**Affiliations:** 1 Nantes Université, Université de Tours, INSERM, MethodS in Patients-Centered Outcomes and HEalth Research, SPHERE, F-44000 Nantes, France; 2 Nantes Université, CHU Nantes, Methodology and Biostatistics Unit, F-44000 Nantes, France; 3 Integrated Center for Oncology (ICO, Institut de Cancérologie de l’Ouest) - Nantes, Angers, France; 4 Nantes Université, CHU Nantes, Public Health Department, F-44000 Nantes, France

**Keywords:** Posttraumatic growth, Psychometric properties, Psycho-oncology, Breast cancer, Melanoma

## Abstract

**Background:**

The Posttraumatic growth inventory (PTGI) aims to assess the positive psychological changes that individuals can perceive after a traumatic life event such as a cancer diagnosis. Several French translations of the PTGI have been proposed, but comprehensive data on their psychometric properties are lacking. This study aimed to provide a more complete assessment of the psychometric properties of one of the most used PTGI translations in early-stage breast cancer and melanoma patients.

**Methods:**

A sample of 379 patients completed the PTGI two years after their cancer diagnosis. A confirmatory analysis was first performed to determine whether the initial five-factor structure of the PTGI was adequate for this French version. As issues were identified in the translation and in the questionnaire structure, we performed an exploratory analysis to determine the most suitable structure for this questionnaire. Validity and reliability of the evidenced structured were then assessed.

**Results:**

The exploratory analysis evidenced a four-factor structure close to the initial structure: four of the five initial domains were recovered, and items from the unrecovered domain were split into the other domains. This new structure showed good internal consistency and acceptable validity.

**Conclusions:**

This study highlights that the process of translation and cross-cultural validation of questionnaires is crucial to obtain valid and reliable psychometric instruments. We advise French psycho-oncology researchers and psychotherapists to (i) use the revised translation of Lelorain et al. (2010) proposed in this manuscript and (ii) use the four scores newly evidenced with a grouping of two response categories.

**Supplementary Information:**

The online version contains supplementary material available at 10.1186/s12874-022-01722-6.

## Introduction

A cancer diagnosis is a traumatic event with many consequences on patients’ life and health in both the short and long terms. The changes caused by cancer in terms of socio-economic and psychological aspects are numerous, with deteriorated health-related quality of life (HRQoL) and well-being, the occurrence of mood disorders such as anxiety and depression, and loss of income due to a job loss or a working time reduction [1–6]. Patients can nonetheless also experience positive psychological changes after cancer. For instance, Sears et al. demonstrated in a qualitative interview-based study that 83% of women diagnosed with early-stage breast cancer found at least one benefit from their experience with cancer [7]. Experiencing positive psychological changes in the aftermath of a struggle with highly challenging life circumstances has been described in the 1990s by Tedeschi and Calhoun as *Posttraumatic growth* (PTG) [8, 9]. PTG can arise, with various intensity, after many different types of events such as war, bereavement, natural disaster, accident, assault or cancer diagnosis [10].

Three forms of positive change have been initially described [9]. First, PTG can manifest itself through perceived changes in self, with increased self-reliance, self-confidence and sense of strength to cope with difficult situations in the future. In addition, individuals facing traumatic events can also change their philosophy of life by redefining their life priorities, growing in spirituality or better appreciating their life. Finally, the experience of trauma can also positively modify interpersonal relationships. Indeed, traumatic events can strengthen bounds between individuals and change the way people interact with each other.

The most widely used measurement tool for assessing PTG is the Posttraumatic Growth Inventory (PTGI) developed by Tedeschi and Calhoun [9]. The PTGI is a self-reported questionnaire composed of 21 items organized into five domains associated with the three above-mentioned forms of positive change. Of note, when the questionnaire was developed, only these three above-mentioned forms of positive change were well identified in the literature; the five domains emerged from the evaluation of the psychometric properties of the PTGI and covered these three expected forms of positive change. Besides, these five domains gave a more refined picture of perceived positive changes. This questionnaire is available in at least 25 languages [10], and its psychometric properties have been evaluated in many populations with various types of traumas. These studies have shown some structural instability, with some authors finding one, two, three, four, or five domains. In the French language, the PTGI has been translated twice. The first translation was performed by Lelorain et al. [11], and the second one was realized by Cadell et al. [12]. Yet, to date, at least three French versions derived from these translations are currently used in France, and comprehensive data regarding their psychometric properties are lacking, notably in cancer patients.

In France, PTG is a growing field in research and clinical care related to life after a trauma [13] such as a cancer diagnosis. Ensuring the validity and reliability of the PTGI questionnaire is essential for researchers to get more insight into the experience of positive change following a cancer diagnosis in clinical research studies. It is also important in clinical care for therapists who need reliable and valid psychometric assessments to identify the resources developed by patients with the aim to adapt their psychological support accordingly.

This study was therefore undertaken to determine the most suitable structure for the French version of the PTGI derived from the translation realized by Lelorain et al. [11] in early-stage breast cancer and melanoma patients. Specifically, this study addresses the structural validity and reliability of the questionnaire through confirmatory and exploratory analyses. It also assesses the concurrent validity with a coping measure and targets the association between PTGI scores and cancer location.

## Material and methods

### The PTGI questionnaire

The PTGI is a self-report questionnaire composed of 21 items organized into five domains: *Relating to Others* (RO, 7 items), *New Possibilities* (NP, 5 items), *Personal Strength* (PS, 4 items), *Spiritual Change* (SC, 2 items), and *Appreciation of life* (AL, 3 items) [9]. All items are scored on a 6-point Likert response format indicating the extent to which the listed changes occurred in the respondents’ lives as a result of an identified trauma. Response categories range from 0 = “*I did not experience this change as a result of my crisis*” to 5 = “*I experienced this change to a very great degree as a result of my crisis*”. Each domain score is computed as the sum of its item responses. A high score on a domain indicates a higher degree of reported positive changes.

In the seminal article on the PTGI, Tedeschi and Calhoun worked on a sample of students at a U.S. university who had experienced a significant negative life event in the past 5 years [9]. They performed a principal component analysis (PCA) on 34 items, followed by a varimax rotation. It produced six factors with eigenvalues greater than one. As only five factors were easily interpretable, they only retained the 21 items loading on these factors. They then performed a second PCA followed by a varimax rotation on these 21 items. It produced five factors with eigenvalues greater than one, identical to those found with 34 items. These factors are the one mentioned above (i.e., *Relating to Others*, *New Possibilities*, *Personal Strength*, *Spiritual Change*, and *Appreciation of Life*). This analysis yielded high factor loadings (0.59 to 0.85), except for two items (item 1, factor loading = 0.50: “*I changed my priorities about what is important in life*”*,* and item 12, factor loading = 0.54: “*I am better able to accept the way things work out*”). The internal consistency of the five factors that emerged was acceptable (Cronbach’s alpha coefficients between 0.67 and 0.85), as was the internal consistency of all items (Cronbach’s alpha coefficient equal to 0.90). In addition, corrected item-total PTGI correlations (the correlation of each item with the total score across all remaining 20 items) ranged from 0.35 to 0.63. Pearson’s correlations among factors ranged from 0.27 to 0.52, and correlations of factors with the PTGI total score ranged from 0.62 to 0.83. Test-retest reliability was assessed over 2 months and seemed globally acceptable. Since then, the factorial structure of the PTGI has been challenged, with some studies finding one, two, three, or four factors [14–20]. Of note, some of these studies used exploratory factor analyses instead of confirmatory factor analyses (CFA), which is the recommended method for confirming a hypothesized factor structure.

Finally, Tedeschi and Calhoun did not find a significant relationship between time elapsed since the negative life event (ranging from less than 6 months to more than 4 years) and the PTGI total score [9], but inconsistent findings have been found in the literature [10]. For instance, longitudinal studies in psycho-oncology showed that PTG could rapidly occur after a cancer diagnosis and then remain stable or increase over almost 2 years [21–23]. At last, sex differences are mostly consistent among studies, with women usually reporting slightly higher PTGI scores than men [10].

### French versions of the PTGI

In the French language, the PTGI has been translated twice. The first translation was proposed by Lelorain et al. [11], and the second one was realized by Cadell et al. [12]. To date, at least three French versions are currently used in France:

- Version 1: The original translation realized by Lelorain et al. [11]. In the literature, no information is available on the translation process used. We learned from the authors that they translated the questionnaire into French and that a native English-speaking professional performed a back-translation. However, we do not know whether any discrepancies were highlighted and whether they were accounted for. Of note, no content validity assessment has been performed. This French version has been adapted for cancer patients who were asked to rate items from 0 = “*Not at all*” to 5 = “*Totally*” to indicate whether cancer caused the change mentioned in the item.
- Version 2: A revised version that appeared in French study protocols in 2010 (following the work of Lelorain et al. [11]). This version shows slight adaptations in wording compared to the original translation (in total, changes in the wording have been made on three items over 21). These changes in wordings from version 1 are addressed in detail in supplementary material (Appendix A).
- Version 3: The translation performed by Cadell et al. [12]. To translate the PTGI from English to French, Cadell et al. used a professional translator. This French version was then independently back-translated into English by another certified translator and no differences were highlighted. The content was validated independently by a native French speaker. Of note, these authors justified their choice to re-translate the PTGI in French because they found no published version of the translation realized by Lelorain et al. [11]. Their French wording of the items are available in their manuscript [12].

Table 1 summarizes the information available on these different versions in the literature. It synthesizes the psychometric properties of each version and lists the studies where they are used as a measurement tool.

**Table 1:** Overview of the available information regarding the three different French versions of the 21-item posttraumatic growth inventory

PTGI French versions	Availability of data on psychometric properties	Reported statistical analyses						French studies involving the version
		Cronbach’s alpha	Inter-item correlation	CFA + Goodness of fit	Convergent validity	Divergent validity	Additional results	
**Version 1:** Original translation developed by Lelorain et al.	See Lelorain et al. [11] **Sample**: *N* = 307 Long term Breast cancer survivors (France)	RO: 0.85 NP: 0.86 SC: 0.83 AL: 0.81 PS: 0.79 Total PTGI (21 items): 0.93	Not available	Hierarchical CFA with 5 first-order factors (one for each domain) and one second-order factor (global PTG) The model did not fit their data well	Not avaible	Not available	No	- Three studies used this version to assess PTG in breast cancer patients [11, 24, 25] - One study used this version to validate the French translation-adaptation of the impact of cancer questionnaire version 2 (VALIOC) [26] - One study used this version to assess PTG in survivors of intimate partner violence [13]
**Version 2:**Revised version based on the translation of Lelorain et al.	No	Not available	Not available	Not available	Not available	Not available	No	- Three studies used this version to assess PTG in cancer survivors: ELCCA [27], VICAN [28], and EPICURE [29] - One study used this version to assess PTG in cancer patients and health care workers during Covid-19 pandemic (PAPESCO-19) [30] - One study used this version to assess PTG in renal failure patients waiting for first kidney transplantation (PreKitQol) [31]
**Version 3:**Translation of Cadell et al.	See Cadell et al. [12] **Sample: ***N* = 10 Bereaved caregivers (Canada) + *N* = 37 Parents caring for a child with a life-limiting illness (Canada)	RO: 0.85 NP: 0.83 SC: 0.86 AL: 0.64 PS: 0.34 (0.77 when omitting item 12^a^) Total PTGI (21 items): 0.87	Within each domain, all items were significantly correlated, except for item 12^a^	Five unidimensional CFAs (one per domain) + One CFA on the five mean scores to the domains *Remark: No multidimensional CFA combining the five domains and the associated items was performed*	All standardized factor loadings from the five CFAs were above 0.5 All standardized factor loadings from the CFA performed on the mean scores were above 0.5 (except for AL score)	Not available	Dimensionality was explored and reported in terms of *discriminant validity*. It was assessed for each pair of domains by comparing the chi-square statistic of two CFAs: 1) A CFA where the correlation between the two factors is constrained to be equal to 1 2) A CFA where the correlation is freely estimated	- One study used this version to assess PTG after a hematopoietic stem-cell transplantation [32] - One study used this version to assess PTG in survivors of a diffuse large B-cell lymphoma [33]

*PTGI* Posttraumatic growth inventory, *PTG* Posttraumatic growth, *RO* Relating to others, *NP* New possibilities, *PS* Personal strength, *SC* Spiritual change, *AL* Appreciation of lie, *N* Sample size, *CFA* Confirmatory Factor Analysis

^a^Item12: “*I am better able to accept the way things work out*”, Cadell French translation: “*J’accepte plus facilement la tournure que prennent les événements*”

On the one hand, the psychometric properties of the two first versions (versions 1 and 2) have never been comprehensively evaluated. Indeed, the only available information regarding the original translation is that a hierarchical CFA with five first-order factors (RO, NP, PS, SC, and AL) and one second-order factor (global PTG) did not fit data from a sample composed of 307 French women who had recovered from breast cancer for at least 5 years [11]. Nonetheless, all five domains and all 21 items showed good internal consistency [11]. After further investigation, the performed CFA might have been too restrictive as no correlation between the error terms was allowed. In addition, no information on convergent and divergent validity is available. As for the version derived from the original translation of Lelorain et al. [11], no information is currently available. To the best of our knowledge, these two versions are the ones that are mainly used in France.

On the other hand, Cadell et al. validated their own French version of the PTGI on a small Canadian sample of French-speaking caregivers (combining 10 bereaved HIV/AIDS caregivers and 37 parents caring for a child with a life-limiting illness) [12]. All domains (except one, i.e., Personal strength) showed Cronbach alpha coefficients greater than 0.6 (the threshold used by the authors as providing evidence supporting reliability for exploratory studies). CFAs performed on each of the five domains indicated satisfactory evidence of convergent validity based on factor loading values. Of note, no multidimensional confirmatory factor analysis combining the five domains and their related items was performed. Dimensionality was also explored using CFAs and reported in terms of *discriminant validity*; the five domains demonstrated a high discriminant validity, indicating that they represented distinct constructs.

In brief, data on the psychometric properties of both the French translation realized by Lelorain et al. [11] (version 1) and the revised version (version 2) are too scarce to rule on the validity of these questionnaires in patients facing cancer. In addition, results emphasized by Cadell et al. [12] may not be transposable to a French population for several reasons. First, French-speaking Canadians may not be representative of French people due to cultural differences. Besides, caregivers and patients experiencing a major health event (such as cancer) might perceive the PTG construct and PTGI questionnaire differently [15, 34]. Finally, the translation of Cadell et al. [12] is quite different from the other two versions with regard to the formulation of the items.

### Patients sample

To assess the psychometric properties of the revised French version of the PTGI (version 2), we used the data collected within the ELCCA cohort (ClinicalTrials.gov Identifier: NCT02893774) [35]. ELCCA is a longitudinal study conducted in France on patients recently diagnosed with early-stage breast cancer or melanoma at the Nantes Cancerology Institute (for breast cancer patients) and the Department of Onco-Dermatology of Nantes University Hospital (for melanoma patients). It was approved by an ethical research committee (“Comité de Protection des Personnes”). The objective of ELCCA was to study socioeconomic, psychological and HRQoL changes following a breast cancer or a melanoma within the months and years following the cancer diagnosis. Breast cancer and melanoma were chosen as they have a similarly good prognosis when detected early. Nevertheless, health care is very different for these two cancer locations. On the one hand, breast cancer treatments are generally invasive with major surgery and therapies such as radio-, chemo- and hormonal therapies. On the other hand, melanoma patients experience a minor surgery possibly followed by immunotherapy [35]. Hence, the different nature of the treatments could interfere with the perceived severity of the disease, and by extension, with the changes that patients may experience.

After the diagnosis, patients were informed about the study by an oncologist and were invited to sign an informed consent agreement. Participants were asked to complete a series of questionnaires at different measurement occasions: 1, 6, 12, 24, 48, and 60 months post-diagnosis. We chose to analyze the data from the 4th measurement occasion (i.e. 2 years post-diagnosis) to allow PTG to take place and allow patients sufficient time to report it [10]. In addition to questionnaires, patients had to report socioeconomic and clinical information. They completed the booklet of questionnaires at home or during a follow-up visit.

### Measures

At each measurement occasion, patients were asked to complete a series of questionnaires that included the PTGI (version 2: revised French version based on Lelorain et al. translation) [9, 11] and the Brief COPE [36, 37]. Of note, the Brief COPE is an abbreviated version of the COPE [38]. It contains 14 subscales (composed of two 4-point Likert items each) aiming to assess the coping strategies used by the patients (active coping, planning, using instrumental support, using emotional support, venting, behavioral disengagement, self-distraction, self-blame, positive reframing, humor, denial, acceptance, religion, and substance use). Subscale scores are computed as the sum of the item responses, with a higher score indicating a higher use of a given strategy to deal with stressful life events.

### Statistical analysis

#### Confirmatory analysis

To determine whether the initial five-factor structure of the PTGI was suitable for the French version used in the ELCCA study, we conducted a confirmatory analysis aiming to inform its structural validity and reliability.

First, we conducted a confirmatory factor analysis (CFA) with oblique factors based on the initial five-factor structure of the PTGI using maximum likelihood estimation [39, 40]. The chi-square statistic used to assess the goodness of fit was corrected using Satorra-Bentler adjustment to obtain results robust to non-normality (item responses being in an ordinal format with a 6-point response scale). Good (or acceptable) fit was indicated by the following criteria: RMSEA ≤0.05 (≤0.08), CFI ≥ 0.95 (≥0.90) and SRMR ≤0.05 (≤0.10). A hierarchical CFA was also conducted to explore a second-order factor structure representing global PTG.

We assessed the item-level convergent validity of each domain to ensure that the items correlated well with their hypothesized domain. It was evaluated by examining the Spearman’s item-rest correlations (i.e., the Spearman’s correlation coefficient between the item score and the domain score computed without the item). Item-level convergent validity was considered good (or acceptable) when 100% (respectively 95%) of the items from a given domain had an item-rest correlation greater than 0.4. Item-level divergent validity was also evaluated to ensure that items were more correlated with their own (hypothesized) domain than with the other domains of the scale. It was assessed by comparing each Spearman’s item-rest correlation coefficient (computed between a given item and its hypothesized domain) with Spearman’s correlation coefficients computed between the studied item and the other domains*.* The item-level divergent validity was considered good (or acceptable) when 100% (respectively 95%) of the items of the questionnaire were more correlated with their hypothesized domain than with the other domains. Correlations between PTGI domains were also examined using Spearman’s correlation coefficients. They were expected to be moderately to highly correlated with one another as found in previous validation studies of the PTGI in cancer patients [41, 42].

Internal consistency of the PTGI domains was assessed using the Cronbach’s alpha coefficient *α* [43]. Domains were considered reliable if α > 0.7. We also used backward Cronbach alpha curves to determine whether domains were unidimensional or not [44]. These curves were obtained for each domain by: (1) Computing the Cronbach’s alpha coefficient over all items of the considered domain, (2) Removing items one by one until there are only two items left. At every successive step, the removed item is the one that left the remaining set of items with the maximum Cronbach’s alpha. If the set of items is not unidimensional, increases in the Cronbach’s alpha coefficient will be observed after item removal. In addition, the Loevinger’s coefficients (H and H_i_) in relation to the domains and to the items, respectively, were used to evaluate the homogeneity of the domains (H > 0.3 indicating a high degree of homogeneity) and to determine whether a given item *i* was consistent with its domain (verified if H_i_ > 0.3) [45, 46].

Of note, personal mean score imputations were realized in case of partial missing data in one or several domains. Within each of the five domains, missing data were imputed by the personal mean score if the number of non-missing items was higher than half the number of items comprising that domain.

#### Exploratory analysis

In case of unsatisfying results, we planned to conduct a clustering of variables around latent components (CLV) analysis to identify the most optimal structure for the French version of the PTGI.

CLV is an exploratory analysis which has been developed by Vigneau and Qannari [47], and aims to identify unidimensional and disjointed sets of items. To prevent the imputation performed during the confirmatory analysis from possibly favoring the emergence of the five initial factors,^1^ we planned to:

- Step 1: Conduct the CLV analysis on the raw data (not imputed by the personal mean score)
- Step 2: Impute missing data using the personal mean score based on the structure evidenced by the CLV analysis during step 1
- Step 3: Ensure that the evidenced structure remains stable by re-running the CLV analysis on this newly imputed data set

All analyses already described for the confirmatory analysis were performed to assess the structural validity and reliability of a new structure potentially evidenced by the CLV analysis (i.e. CFA, item-level convergent and divergent validity, and reliability).

#### Concurrent validity and comparison of PTGI scores according to cancer location

We explored the concurrent validity and compared the PTGI scores according to cancer location based on the most optimal structure for the French version of the PTGI studied in this manuscript (structure evidenced by either the confirmatory factor analysis or the exploratory analysis).

Concurrent validity was evaluated by assessing the plausibility of a priori assumptions about patterns of association between the PTGI scores and the Brief COPE scores. Tedeschi and Calhoun asserted that PTG was both a process and an outcome [48]. Specifically, PTG could be a strategy for coping, managing and surviving trauma just after it occurs (i.e., a process), and then turn into an outcome at a later time, as positive changes can be expressed after a challenging experience [10]. Hence, we expected positive correlations between PTGI scores and the following domains of the Brief COPE: active coping, planning, using instrumental support, using emotional support, venting, positive reframing, humor, and acceptance. The hypothesized domain comprising the item 5 and 18 (related to a Spiritual Change according to Tedeschi and Calhoun’s theoretical rationale [9]) was also expected to be strongly correlated with religious coping.

The association between the PTGI scores and the cancer location were examined using Mann-Whitney tests. PTGI scores were expected to be higher for breast cancer patients, as breast cancer patients may experience more social support than melanoma patients [35, 49] and since previous research suggested that experience sharing and social support are associated with PTG [50]. To withdraw the gender effect in the comparison of melanoma (both women and men) and breast cancer patients (only women), we restricted this score comparison to the data collected in women.

All analyses were performed with Stata 16 (*Stata Statistical Software: Release 16*. College Station, TX: StataCorp LLC). The Stata module used to perform CLV is available from the Statistical Software Components archive [51]. The French version of the PTGI evaluated in this manuscript (i.e., the version 2 that derived from Lelorain et al. translation [11]) is available in the supplementary materials (see the Appendix B).

## Results

### Sample characteristics

At the 4th measurement occasion (i.e., 2 years after their cancer diagnosis), 380 participants sent back the PTGI questionnaire. Among them, one patient with breast cancer only responded to one item, she was excluded from the analysis. Of the 379 remaining participants, 299 (79%) had breast cancer and 80 (21%) had melanoma. They were aged between 21 and 73 years, with an average age of 54.8 (SD = 9.1 years). All participants with breast cancer were women. Among melanoma patients, 32 were women (40%) and 48 were men (60%). Most of them lived in couple (81%). A total of 361 out of 379 (95%) individuals had a complete PTGI (i.e., no missing data in their responses). Item-level missing data rates ranged from 0.3 to 1.8% (this latter rate being reached by item 18: “*I have a stronger religious faith*”).

### Confirmatory analysis

#### Confirmatory factor analysis

The confirmatory factor analysis based on the initial five-factor structure of the PTGI (with oblique factors) indicated an acceptable fit suggested by the goodness of fit criteria (with Satorra-Bentler correction): χ^2^(179, *n*= 369) = 507.4, *p* < 0.001, χ^2^/df = 2.8, RMSEA = 0.071, CFI = 0.909, SRMR = 0.048. All standardized factor loadings were greater than 0.55; they are given in Table 2. High covariances ranging from 0.76 to 0.90 were observed among four of the five factors: *Relating to others*, *New possibilities*, *Personal strength* and *Appreciation of Life*. When the *Spiritual change* factor was involved, covariance did not exceed 0.43. Of note, adding a second-order factor (i.e., global PTG) through a hierarchical CFA did not improve the model fit: χ^2^(184, *n*= 369) = 521.5, *p* < 0.001, χ^2^/df = 2.8, RMSEA = 0.071, CFI = 0.906, SRMR = 0.050. In this hierarchical CFA, the factor loading associated with the *Spirituality change* factor was low (0.42). Together with the moderate covariances observed between the *Spiritual change* factor and the other factors within the oblique CFA, these results indicate that the PTGI should be considered a multidimensional scale. Hence, we did not consider a total score computed over the 21 items in the following. Finally, adding covariance between the error terms of two items from the same domain (based on large modification indices) did not really improve model fit.

**Table 2:** Standardized factor loadings of the 21 items of the posttraumatic growth inventory obtained after the oblique confirmatory factor analysis based on the initial five-factor structure

	Standardized factor loadings for each factor				
PTGI items	RO	NP	PS	SC	AL
***Relating to others (RO)***					
6. I more clearly see that I can count on people in times of trouble *Je vois mieux que je peux compter sur les autres en cas de problème*	0.56	–	–	–	–
8. I have a greater sense of closeness with others *Je me sens plus proche des autres*	0.81	–	–	–	–
9. I have a greater willingness to express my emotions *Je suis plus enclin(e) à exprimer mes émotions*	0.76	–	–	–	–
15. I have greater compassion for others *J’ai plus de compassion pour les autres*	0.62	–	–	–	–
16. I put more effort into my relationships *J’investis plus mes relations aux autres*	0.81	–	–	–	–
20. I learned a great deal about how wonderful people are *Je vois plus le bon côté des gens*	0.77	–	–	–	–
21. I better accept needing others *J’accepte mieux le fait d’avoir besoin des autres*	0.71	–	–	–	–
***New possibilities (NP)***					
3. I developed new interests *Je me suis intéressé(e) à de nouvelles choses*	–	0.77	–	–	–
7. I established a new path for my life *J’ai donné une nouvelle direction à ma vie*	–	0.77	–	–	–
11. I’m able to do better things with my life *Je fais de ma vie quelque chose de meilleur*	–	0.83	–	–	–
14. New opportunities are available which wouldn’t have been otherwise *De nouvelles opportunités sont apparues*	–	0.67	–	–	–
17. I’m more likely to try to change things which need changing *J’essaie davantage de changer les choses qui ont besoin d’être changées*	–	0.72	–	–	–
***Personal strength (PS)***					
4. I have a greater feeling of self-reliance *J’ai acquis plus confiance en moi*	–	–	0.75	–	–
10. I know better that I can handle difficulties *Je suis davantage capable de gérer des situations difficiles*	–	–	0.77	–	–
12. I am better able to accept the way things work out *J’accepte mieux la façon dont les choses se passent*	–	–	0.78	–	–
19. I discovered that I’m stronger than I thought I was *J’ai découvert que je suis plus fort(e) que ce que je pensais*	–	–	0.68	–	–
***Spiritual change (SC)***					
5. I have a better understanding of spiritual matters *J’ai développé une certaine spiritualité*	–	–	–	0.94	–
18. I have a stronger religious faith *J’ai une foi religieuse plus grande*	–	–	–	0.83	–
***Appreciation of life (AL)***					
1. I changed my priorities about what is important in life *J’ai changé de priorités dans la vie*	–	–	–	–	0.59
2. I have a greater appreciation for the value of my own life *J’apprécie plus ma vie à sa vraie valeur*	–	–	–	–	0.79
13. I can better appreciate each day *J’apprécie plus amplement chaque jour de ma vie*	–	–	–	–	0.83

The wording of the items in the French version of the PTGI that we studied (version 2) are shown in italics

#### Item-level convergent-divergent validity

All items had a Spearman’s item-rest correlation with their hypothesized domain greater than 0.4, confirming the convergent validity. However, criterion for divergent validity was not met; indeed only 17 items over the 21 (i.e., 81%) were more correlated with their hypothesized domain than with the other domains. Specifically, all items from the *Relating to others*, *New possibilities,* and *Spiritual Change* domains were more strongly correlated with their hypothesized domain than with other ones. However, some items from the *Personal strength* and *Appreciation of Life* domains were divergent:

- Item 12 (*Personal Strength* domain): “*I am better able to accept the way things work out*” was more strongly correlated with the *New possibilities* and *Appreciation of life* domains (Spearman’s correlation coefficients were 0.62 and 0.61, respectively) than with the *Personal strength* domain (Spearman’s item-rest correlation was 0.60). Nevertheless, the difference between the correlations remained small.
- Item 1 (*Appreciation of life*): “*I changed my priorities about what is important in life*” had a Spearman’s correlation coefficient of 0.59 with the *New possibilities* domain while its Spearman’s item-rest correlation with the *Appreciation of life domain* was 0.47.
- Item 13 (*Appreciation of life*): “*I can better appreciate each day*” correlated more with two other domains: *New possibilities* and *Personal strength* (correlation coefficients were respectively 0.61 and 0.64) than with *Appreciation of life* (item-rest correlation of 0.55).

#### Correlation between the five PTGI domains

Spearman’s correlation coefficients between the *Relating to others*, *New possibilities*, *Personal strength* and *Appreciation of life* domains were high (ranging from 0.62 to 0.72). Spearman’s correlation coefficients between the *Spiritual change* domain and the other domains were small to moderate (between 0.25 and 0.36).

#### Reliability

**Fig. 1:**
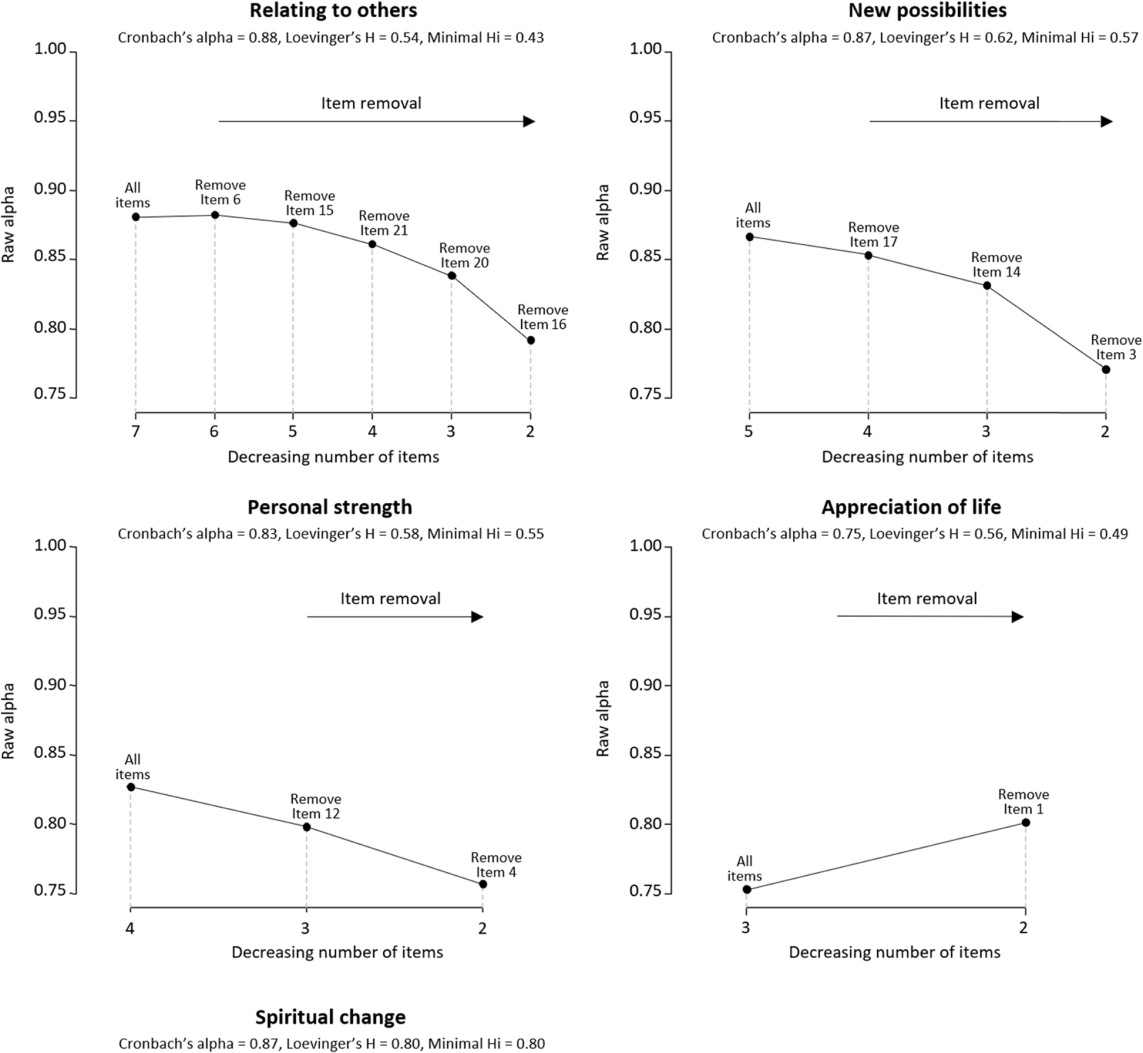
Cronbach’s and Loevinger’s coefficients for the five initial domains and backward Cronbach alpha curves for the four domains composed of three items or more *Figure notes: - Minimal Hi: Minimal Loevinger’s coefficient at the item level over all items of the domain. - The Spiritual Change domain is composed of two items, hence no backward Cronbach alpha curve can be drawn*

All domains showed good internal consistency with Cronbach’s alpha coefficients ranging from 0.75 to 0.88. Loevinger coefficients related to the domains (H) varied between 0.54 and 0.80, indicating acceptable homogeneity for all domains. At the item level, all items exhibited Loevinger coefficients greater than 0.3. Hence, all items seemed to be consistent within their domain. All coefficients regarding reliability are given in Fig. 1 alongside the backward Cronbach alpha curves. Of note, a dimensionality issue appeared within the *Appreciation of Life* domain: the associated backward Cronbach alpha curve increased when removing item 1. It indicated that this domain might not be unidimensional.

#### Summary

Based on statistical results, the studied French version of the PTGI showed mixed psychometric properties. Indeed, the fit of the CFA was acceptable and the convergent validity was good. However, the *Appreciation of Life* domain seemed problematic: two items over three correlated more with other domains, and the alpha curve emphasized a dimensionality issue.

We also noticed several translational issues. For instance, in the wording of French items, the notion of ability has completely disappeared for some items (e.g.*,* items 11 and 12). In addition, the wording of the French response categories is different. Indeed, instead of describing a degree of change (as in the initial version of Tedeschi and Calhoun [9]), Lelorain et al. chose to use the following response categories: 0 = “*Not at all*”, 1 = “*Very few*”, 2 = “*Few*”, 3 = “*A little*”, 4 = “*A lot*”, and 5 = “*Totally*” [11]. Yet, in the French language, the response categories 1 and 2 are hardly differentiable, which might confuse the respondents.

To overcome these issues, we grouped the response categories 1 and 2, and conducted an exploratory study to identify the most optimal structure for this French version of the PTGI in terms of validity and reliability.

### Exploratory analysis

#### Clustering of variables around latent components

**Fig. 2:**
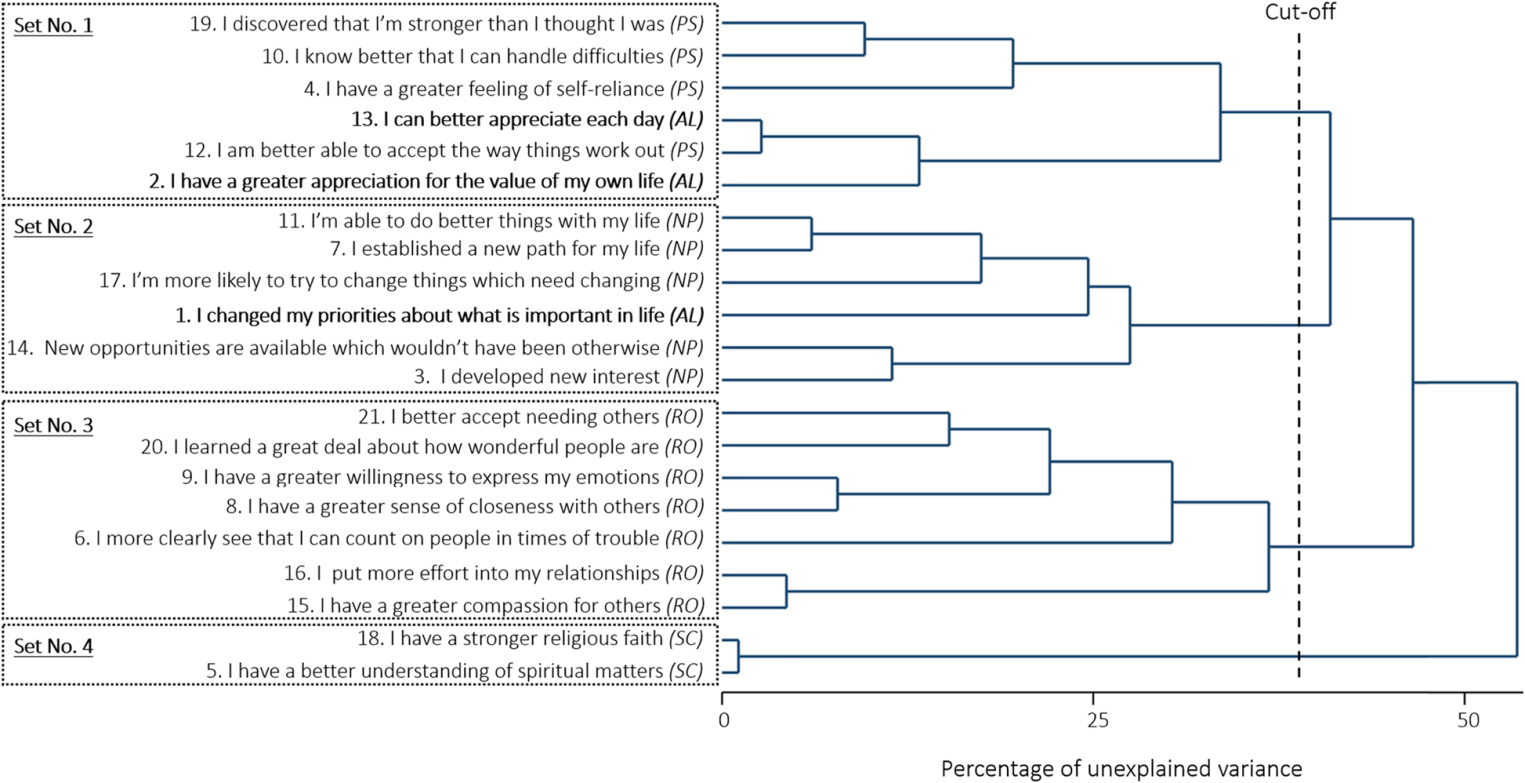
Dendrogram of the Clustering around Latent Variable analysis. Initial domains are indicated in brackets. New unidimensional sets of items are emphasized by boxes *Figure notes: PS: Personal strength, AL: Appreciation of life, NP: New possibilities, RO: Relating to others, SC: Spiritual change*

The CLV analysis was performed on the raw data (not imputed, *n* = 361) where response categories 1 and 2 were grouped beforehand. The analysis led to four unidimensional sets of items that were close to the initial five domains of the PTGI (the dendrogram is given in Fig. 2):

- **Set of items No. 1 [*Personal capacities*]:** All items from the *Personal strength* domain plus two items from the *Appreciation of Life* domain (items 2 “*I have a greater appreciation for the value of my own life*” and 13 “*I can better appreciate each day*”)
- **Set of items No. 2 [*New life direction*]:** All items from the *New possibilities* domain plus one item from the *Appreciation of life* domain (item 1 “*I changed my priorities about what is important in life*”)
- **Set of items No. 3 [*Relating to others*]:** All items from the *Relating to others* domain
- **Set of items No. 4 [*Spiritual change*]:** All items from the *Spiritual Change* domain

Hence, four of the five initial domains were recovered. The fifth domain (i.e., *Appreciation of life*) was not. The three items composing it have been separated; two of them were grouped with the items from the *Personal strength* domain, and the other one was grouped with the items from the *New possibilities* domain. We labelled the sets of items No. 1 and No. 2 *Personal capacities* and *New life direction*, respectively, as these labels better reflected the items composing these new domains and the groupings that have been made. In this newly evidenced four-factor structure, the sum score goes from 0 to 24 for the sets of items No. 1 and No. 2 (*Personal capacities* and *New life direction, 6 items each*), from 0 to 28 for the set No. 3 (*Relating to others, 7 items*), and from 0 to 8 for the set No. 4 (*Spiritual change, 2 items*). Of note, the dendrogram remained the same when re-running the CLV analysis after the missing data imputation by the personal mean score based on this new four-domain structure.

#### Confirmatory factor analysis

The confirmatory factor analysis based on the newly evidenced four-factor structure indicated an acceptable fit suggested by the goodness of fit criteria (with Satorra-Bentler correction): χ^2^(183, *n*= 371) = 524.0, *p* < 0.001, χ^2^/df = 2.9, RMSEA = 0.071, CFI = 0.908, SRMR = 0.048. All standardized factor loadings were above 0.55, they are given in the supplementary materials (Appendix C). Strong covariances among *Relating to others*, *New life direction* and *Personal capacities* factors were again observed (they were all above 0.80). Covariances involving the *Spiritual change* factor were lower and did not exceed 0.41.

#### Item-level convergent-divergent validity

All items had an item-rest correlation with their hypothesized domain greater than 0.4, confirming the convergent validity. In addition, all items exhibited larger correlation coefficients with their domain than with the other ones (except item 11 “*I’m able to do better things with my life*”, which correlated slightly more with the *Personal capacities* domain than with the *New life direction* domain: Spearman’s correlation coefficients were 0.73 versus 0.69). This result indicated an acceptable divergent validity.

#### Correlation between the four new PTGI domains

Spearman’s correlation coefficients among the *Relating to others*, *New life direction*, and *Personal capacities* domains were high (ranging from 0.68 to 0.72). Spearman’s correlation coefficients between the *Spiritual change* domain and the other domains were moderate (between 0.30 and 0.33).

#### Reliability

**Fig. 3:**
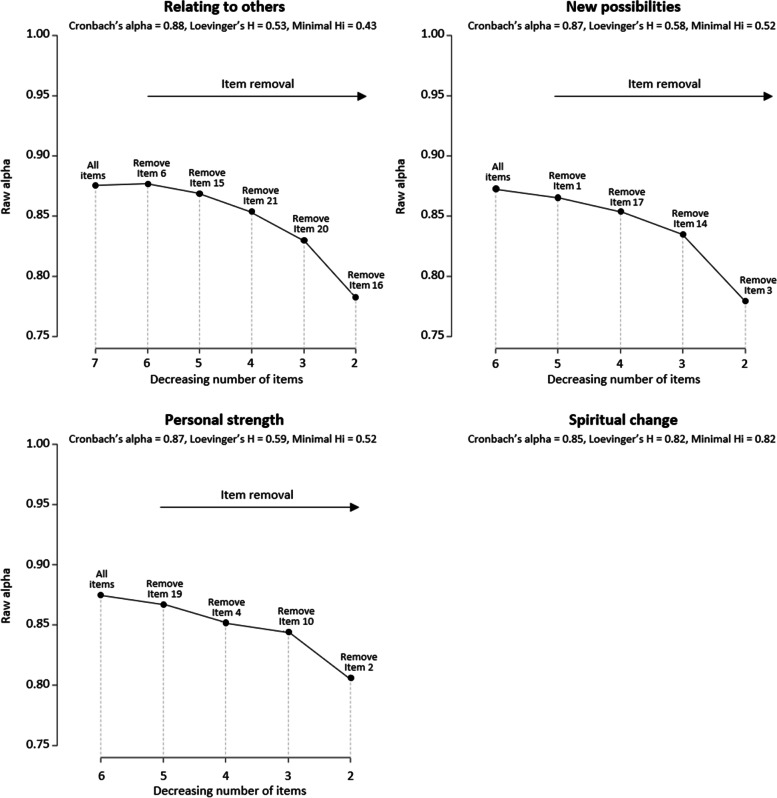
Cronbach’s and Loevinger’s coefficients of the four new domains evidenced by the Clustering around Latent Variable analysis and backward Cronbach alpha curves for the three domains composed of three items or more *Figure notes: Minimal Hi: Minimal Loevinger coefficient at the item level over all items of the domain. The Spiritual Change domain is composed of two items, no backward Cronbach alpha curve can be drawn*

All domains showed good internal consistency and homogeneity as Cronbach alphas were all greater or equal to 0.85 and Loevinger coefficients were greater than 0.4 (values for each domain are given in Fig. 3). In addition, backward Cronbach alpha curves did not evidence dimensionality issues since all curves decrease with item withdrawal (see the Fig. 3).

#### Summary

This new four-factor structure shows better psychometric properties than the five-factor structure. Hence, the rest of the analyses (i.e., concurrent validity and comparison of PTGI scores according to cancer location) will be performed on this structure comprising four domains.

### Concurrent validity

Spearman’s correlation coefficients between the new PTGI scores and the Brief Cope scores are given in Table 3, part (a). As expected, we observed positive correlations between the new PTGI domains *Relating to others*, *New life direction* and *Personal capacities* and the following domains of the Brief COPE: active coping, planning, using instrumental support, using emotional support, venting, positive reframing, acceptance, and humor. Of note, correlations between acceptance, emotional support and the three above-mentioned domains of the PTGI were nonetheless small. The strong link between religious coping and the *Spiritual change* domain was also confirmed, but religious coping was actually positively correlated with all the PTGI domains.

**Table 3:** (a) Correlations between the new PTGI scores and the Brief Cope scores and (b) associations between the new PTGI scores and the cancer location

	Relating to others	New life direction	Personal capacities	Spiritual Change
*(a)* *Correlation with Brief Cope scores (Spearman’s correlation coefficients r)*				
Positive reframing	0.37^*^	0.40^*^	0.46^*^	0.25
Active coping	0.35^*^	0.41^*^	0.39^*^	0.15
Planning	0.34^*^	0.38^*^	0.30^*^	0.19
Humor	0.35^*^	0.32^*^	0.36^*^	0.03
Acceptance	0.22	0.21	0.31^*^	0.08
Self-distraction	0.17	0.24	0.15	0.08
Venting	0.37^*^	0.36^*^	0.28	0.11
Instrumental support	0.36^*^	0.35^*^	0.22	0.15
Emotional support	0.23	0.24	0.11	0.13
Religion	0.31^*^	0.28	0.22	0.75^*^
Self-blame	0.18	0.20	0.02	0.18
Denial	0.06	0.03	0.02	0.07
Behavioral disengagement	−0.07	−0.10	−0.13	0.01
Substance use	− 0.08	− 0.04	−0.09	− 0.04
*(b)* *Association with cancer location (median of the sum scores for each domain and IQR)*				
Women with breast cancer (*n* = 299)	14.0 [IQR = 8.0]	10.0 [IQR = 7.0]	13.0 [IQR = 6.0]	1.0 [IQR = 3.0]
Women with melanoma (*n* = 32)	14.0 [IQR = 8.5]	8.5 [IQR = 7.0]	10.5 [IQR = 7.5]	0.0 [IQR = 1.5]
*p*-value ^a^	0.321	0.030	0.046	0.034

*IQR* Interquartile range, *n* sample size

^*^: |*r*| ≥ 0.3 and *p*-value <0.05

^a^ Mann-Whitney tests

### PTGI scores according to the cancer location

As expected, breast cancer women showed significantly higher PTGI scores than melanoma women with a significance level of 5%, except for the *Relating to others* domain for which no significant difference was found. Description of the PTGI scores according to the cancer location among women is available in Table 3, part (b).

## Discussion

### Main results

In this current study, we first examined the psychometric properties of the French version of the PTGI in early-stage breast cancer and melanoma patients based on the initial five-factor structure [9]. This questionnaire showed mixed psychometric properties. Indeed, while the fit of the CFA was acceptable and the convergent validity was good, the *Appreciation of Life* domain seemed problematic. In addition, we noticed several translational issues in the items wording and in the response categories. Hence, we decided to group two response categories that were hardly differentiable in French language and searched for the optimal structure for this French version of the PTGI in terms of validity and reliability.

Based on an exploratory factor analysis, we evidenced a four-factor structure close to the initial five-factor structure. Indeed, four of the five initial domains were recovered, and the items from the unrecovered domain, i.e. *Appreciation of life*, have been separated; two of them were grouped with the items from the *Personal strength* domain, and the other one was grouped with the items from the *New possibilities* domain. On the one hand, item 1 (“*I changed my priorities about what is important in life*”) was grouped with the items from the *New possibilities* domains. It made sense as patients probably perceived this item as dealing with the changes regarding their life orientation (e.g., things they want or no longer want to dedicate time to, things they want to change, and paths they want to follow) making this item close to items from the *New possibilities* domain. We labelled this new set of items *New life direction*, as it reflects the novelty and being active (as opposed to passive) regarding life orientation. On the other hand, items 2 (“*I have a greater appreciation for the value of my own life”*) and 13 (“*I can better appreciate each day*”) were grouped with the items from the *Personal Strength* domain. More precisely, we can observe in the dendrogram of the CLV analysis (Fig. 2) that these items are rapidly grouped with item 12 (“*I am better able to accept the way things work out*”). This merging might be explained by the strong correlation between items 2, 12, and 13 (ranging from 0.6 to 0.7). A possible explanation for these associations is that acceptance (targeted by item 12) has been shown to promote well-being (targeted by items 2 and 13) as reported in papers dealing with Acceptance and Commitment therapy [52–54]. We labelled this new set of items *Personal capacities*, as the confrontation with trauma resulted in the development of emotion regulation strategies.

Regarding the concurrent validity, PTGI scores to the *Relating to others*, *New life direction* and *Personal capacities* domains (based on the new four-factor structure) were positively correlated with positive and emotional copings, that is, positive reframing, active coping, planning, humor, acceptance, venting, using instrumental support and religion. These findings are consistent with our expectations and with literature focusing on cancer patients [11, 21, 55–57]. The strong positive association between religious coping and the *Spiritual change* domain was also confirmed, but religious coping was actually positively correlated with all the PTGI domains. This result is also consistent with literature [58–60]. Besides, except for the correlation between religious coping and *Spiritual change*, the correlations between PTGI and Brief COPE scores were moderate in absolute values. This result is in line with the debate about whether PTG is a coping strategy or not [10].

Finally, when compared with melanoma patients, breast cancer patients showed higher scores for all domains of the PTGI except one (i.e., *Relating to others*), for which no significant difference was evidenced. These results are in line with our expectations. A possible explanation is that breast cancer patients might perceive their disease as more severe than melanoma patients. Indeed, Bourdon et al. found in a qualitative interview-based study that melanoma may be trivialized because it is asymptomatic in its early stages [61]. In addition, research among cancer patients showed that people who perceive their cancer as a traumatic or highly stressful event are more likely to report PTG [62–64].

Of note, we could not compare our results with those obtained by Lelorain et al. [11] or Cadell et al. [12] as the retained structure of the questionnaire was not the same and because the data they provided did not match the analysis performed in our study.

### Limitations and perspectives

Our study has several limitations that can be mentioned. First, inclusion criteria targeted only early-stage cancer patients from two hospital units (one per cancer site) data were collected 2 years after the cancer diagnosis for all patients. This may limit the generalizability of our results. Further validation studies across different cancer diagnoses, with various times since the onset of the disease, and in other French-speaking countries are needed to assess the psychometric stability of the French version of the PTGI (i.e., reproducibility under conditions of limited change). In addition, during Study 2, we performed the CLV analysis and the CFA on the same sample. A CFA using another independent sample should be carried out in the future to investigate structure stability. Finally, the responsiveness of the PTGI to changes throughout remission has not been evaluated, and the ELCCA study design did not allow us to assess the test-retest reliability (as time-laps between two measurement occasions were large).

Regarding the translation into French, the original translation of Lelorain et al. [11] may not have been realized by a professional translator, and although a back translation has been performed, we do not know if discrepancies were evidenced and whether they were accounted for. When we compared the items wording of the French and English versions, we noticed two main discrepancies. First, in the French version, item 5 (which initially tackled the better understanding of spiritual matters in the English version) asks individuals whether they have experienced spiritual growth. Yet, having a better understanding of spirituality refers to spiritual changes that do not necessarily mean that patients grow in spirituality. Indeed, change in spirituality can be either characterized by growth or decline [65, 66]. Second, the reference to *ability* (e.g., *being able to* do something) disappeared in the French wording of some items. For instance, the French translation of item 12 could be back-translated as: “*I better accept the way things work out*”, whereas the English version was “*I am better able to accept the way things* work *out*”. On the one hand, the wording “*I am better able to accept”* may suggest that acceptance could depend on the situation the patient is facing; it provides context-related information. On the other hand, *“I better accept”* is a broader statement that could apply to all situations encountered; it gives more general information. Therefore, due to the disappearance of the reference to ability, professionals using PTGI for psychological support may miss context-related information, whereas this is often the information sought in psychotherapy [67]. Compared to Lelorain et al. [11] translation, the PTGI version we studied presents major changes in wording for two items. First, the second part of item 14 (“*New opportunities are available, which wouldn’t have been otherwise*”), disappeared in the version we studied. In addition, item 20 (English version: “*I learned a great deal about how wonderful people are*”) has been entirely reformulated in the studied version and could be back-translated as “*I see more the good side of people*”. If the notion of goodness in people can be found in both wording, it is much more prominent in the English version. Assessing the impact of these differences in wording could be interesting, but it would require the collection of data on the original translation of Lelorain et al. [11] in the target population. Finally, the wording of the French response categories is different from the original wording of Tedeschi and Calhoun [9] . Indeed, instead of describing a degree of change, the response categories have been translated by Lelorain et al. as follows: 0 = “*Not at all*”, 1 = “*Very few*”, 2 = “*Few*”, 3 = “*A little*”, 4 = “*A lot*”, and 5 = “*Totally*” [11]. As already mentioned, we chose to group response categories 1 and 2 as their French wordings were very close and might confuse respondents. As some discrepancies can be found between the French versions and the original version of Tedeschi and Calhoun [9] (at both the item and response categories levels), we would advise not comparing French scores with non-French speaking countries. Of note, the possibility of such shifts in the items meaning across translations has already been pointed out by Tedeschi et al. [10].

Finally, to avoid the co-existence of several versions, we would also advise publishing the translated questionnaire alongside the final report and/or validation manuscript.

## Conclusion

This study draws a more comprehensive assessment of the psychometric properties of one of the most widely used PTGI French translations in cancer patients. It highlights that the process of translation and cross-cultural validation is crucial and must follow a rigorous methodology to obtain a reliable and valid assessment tool, valuable in both research and psychotherapy. Indeed, the PTGI allows to capture the positive change following a traumatic situation such as a cancer diagnosis and reflect how much an individual has positively re-assessed his/her life values and worldview [68]. This instrument allows relying on a measure of positive change in addition to the anxiety and depression measures and provides objective feedback on psychological support effectiveness. Given the results, we recommend that French professionals in psycho-oncology use the revised translation of Lelorain et al. [11] with five response categories instead of six (see the Appendix D of the supplementary materials) and avoid using the original five scores of the PTGI but rather the four scores proposed in our manuscript.

## Supplementary Information

**Additional file 1.**

## Data Availability

Research data are not shared. The French version of the posttraumatic growth inventory evaluated in the manuscript is available in the supplementary materials (Appendix B) alongside the final version of the questionnaire (Appendix D). Analysis code for this study is available by emailing the corresponding author. Funding: ELCCA was financially supported by the “Ligue Nationale contre le Cancer” and the “Institut pour la Recherche en Santé Publique”. Y. Dubuy received a national grant from the French Ministry of Higher Education, Research and Innovation, and M. Bourdon was funded by the SIRIC ILIAD INCA-DGOS-Inserm 12558 grant.

## Abbreviations

AL: Appreciation of life
CFA: Confirmatory factor analysis
CFI: Comparative fit index
CLV: Clustering of variables around latent components
HIV/AIDS: Human immunodeficiency virus infection and acquired immunodeficiency syndrome
HRQoL: Health-related quality of life
NP: New possibilities
PCA: Principal component analysis
PS: Personal strength
PTG: Posttraumatic growth
PTGI: Posttraumatic growth inventory
RMSEA: Root mean square error of approximation
RO: Relating to others
SC: Spiritual change
SRMR: Standardized root mean squared residual

## Footnotes

1 During the confirmatory analysis, missing data were imputed by the personal mean score based on the initial five-factor structure.
