# Supplementary material for: Posttraumatic growth inventory: challenges with its validation among French cancer patients
Source: BMC Med Res Methodol. 2022 Sep 24;22:246. doi: 10.1186/s12874-022-01722-6 (PMC9508777; doi:10.1186/s12874-022-01722-6)
Supplement: Supplementary file 1 — Additional file 1. [file 12874_2022_1722_MOESM1_ESM.docx]

**Supplementary Materials**

**Manuscript title:**

**Posttraumatic Growth Inventory: challenges with its validation among French cancer patients**

**Appendix A:** Inventory of the wording changes between the French translation realized by Lelorain et al. (PTGI French version 1) and the version that derived from this translation (version 2)

We noticed two French versions of the PTGI equally used in France (see Table 1 of our manuscript):

-  **Version 1 (V1):**The original translation realized by Lelorain et al.

**- Version 2 (V2):** A revised version with slight adaptations in wording compared to the original translation.

The revised version V2 seemed to have appeared in 2010 following the work of Lelorain et al. It first appeared in a French study protocol. Since then, this revised version has been disseminated in France to various research teams without any mention of the changes made to the wording of some items.

We noted changes in the wording of three items when comparing different study protocols involving the PTGI (the remaining 18 items being identical). These wording changes are addressed in Table S1.

| **Item with wording changes** | | **Authors’ comments** |
| --- | --- | --- |
| **13**. | V1 : J’apprécie **davantage** chaque jour de ma vie  V2 : J’apprécie **plus amplement** chaque jour de ma vie | This is a minor change, as the meaning of the item is maintained. |
| **14.** | V1 : De nouvelles opportunités sont apparues, qui ne seraient pas apparues autrement  V2 : De nouvelles opportunités sont apparues, ~~qui ne seraient pas apparues autrement~~ | The end of the item (indicating that the new opportunities would not have appeared without the disease) has been deleted in the version 2. Item from version 1 is the closest to the English version. |
| **20***.* | V1 : J’ai vraiment compris à quel point les gens pouvaient être formidables.  V2 : Je vois plus le bon côté des gens | Item from version 1 is a literal translation of the English version. It is semantically correct, but it may not be adapted to the French culture. The item has been entirely rephrased in version 2, probably to overcome this issue. If the notion of goodness in people can be found in both wording, it is much more prominent in the original version. |

**Table S1:** Inventory of the wording changes observed between the original translation of Lelorain et al. (V1) and the revised version (V2)

**Appendix B:** French version of the posttraumatic growth inventory used in the ELCCA study and evaluated in our manuscript. It corresponds to the revised version (i.e., version 2) derived from the translation of Lelorain et al.
The wording of the items in the English version is indicated in square brackets.


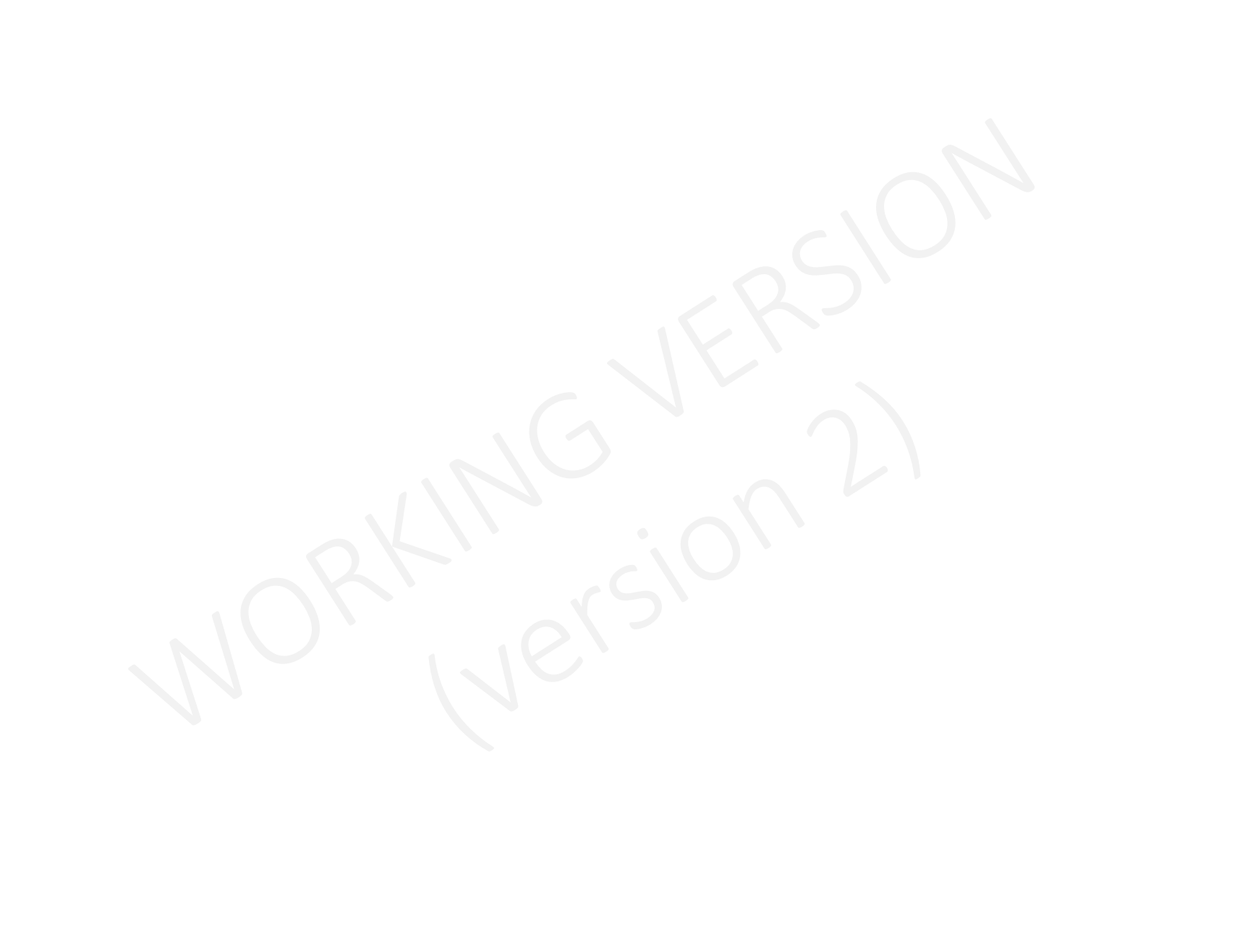
**Inventaire du Développement Post-Traumatique**

| du fait de mon cancer : | pas du tout  0 | très peu  1 | peu  2 | un peu  3 | beaucoup  4 | totalement  5 |
| --- | --- | --- | --- | --- | --- | --- |
| 1. J’ai changé de priorités dans la vie  [I changed my priorities about what is important in life] |  |  |  |  |  |  |
| 2. J’apprécie plus ma vie à sa vraie valeur  [I have a greater appreciation for the value of my own life] |  |  |  |  |  |  |
| 3. Je me suis intéressé(e) à de nouvelles choses  [I developed new interests] |  |  |  |  |  |  |
| 4. J’ai acquis plus confiance en moi  [I have a greater feeling of self-reliance] |  |  |  |  |  |  |
| 5. J’ai développé une certaine spiritualité  [I have a better understanding of spiritual matters] |  |  |  |  |  |  |
| 6. Je vois mieux que je peux compter sur les autres en cas de   problème  [I more clearly see that I can count on people in times of trouble] |  |  |  |  |  |  |
| 7. J’ai donné une nouvelle direction à ma vie  [I established a new path for my life] |  |  |  |  |  |  |
| 8. Je me sens plus proche des autres  [I have a greater sense of closeness with others] |  |  |  |  |  |  |
| 9. Je suis plus enclin(e) à exprimer mes émotions  [I have a greater willingness to express my emotions] |  |  |  |  |  |  |
| 10. Je suis davantage capable de gérer des situations difficiles  [I know better that I can handle difficulties] |  |  |  |  |  |  |
| 11. Je fais de ma vie quelque chose de meilleur  [I’m able to do better things with my life] |  |  |  |  |  |  |
| 12. J’accepte mieux la façon dont les choses se passent  [I am better able to accept the way things work out] |  |  |  |  |  |  |
| 13. J’apprécie plus amplement chaque jour de ma vie  [I can better appreciate each day] |  |  |  |  |  |  |
| 14. De nouvelles opportunités sont apparues  [New opportunities are available which wouldn’t have been otherwise] |  |  |  |  |  |  |
| 15. J’ai plus de compassion pour les autres  [I have greater compassion for others] |  |  |  |  |  |  |
| 16. J’investis plus mes relations aux autres  [I put more effort into my relationships] |  |  |  |  |  |  |
| 17. J’essaie davantage de changer les choses qui ont besoin d'être  changées  [I’m more likely to try to change things which need changing] |  |  |  |  |  |  |
| 18. J’ai une foi religieuse plus grande  [I have a stronger religious faith] |  |  |  |  |  |  |
| 19. J’ai découvert que je suis plus fort(e) que ce que je pensais  [I discovered that I’m stronger than I thought I was] |  |  |  |  |  |  |
| 20. Je vois plus le bon côté des gens   [I learned a great deal about how wonderful people are] |  |  |  |  |  |  |
| 21. J’accepte mieux le fait d'avoir besoin des autres  [I better accept needing others] |  |  |  |  |  |  |

**Appendix C:** Standardized factor loadings from the confirmatory factor analysis based on the newly evidenced four-factor structure

|  | **Standardized factor loadings for each factor** | | | |
| --- | --- | --- | --- | --- |
| **PTGI items** | **RO** | **NLD** | **PC** | **SC** |
| ***Relating to others (RO)*** |  |  |  |  |
| 6. I more clearly see that I can count on people in times of trouble  *Je vois mieux que je peux compter sur les autres en cas de problème* | 0.55 | - | - | - |
| 8. I have a greater sense of closeness with others  *Je me sens plus proche des autres* | 0.82 | - | - | - |
| 9. I have a greater willingness to express my emotions  *Je suis plus enclin(e) à exprimer mes émotions* | 0.75 | - | - | - |
| 15. I have greater compassion for others  *J’ai plus de compassion pour les autres* | 0.63 | - | - | - |
| 16. I put more effort into my relationships  *J’investis plus mes relations aux autres* | 0.80 | - | - | - |
| 20. I learned a great deal about how wonderful people are  *Je vois plus le bon côté des gens* | 0.75 | - | - | - |
| 21. I better accept needing others  *J’accepte mieux le fait d'avoir besoin des autres* | 0.69 | - | - | - |
| ***New life orientation (NLD)*** |  |  |  |  |
| 3. I developed new interests  *Je me suis intéressé(e) à de nouvelles choses* | - | 0.78 | - | - |
| 7. I established a new path for my life  *J’ai donné une nouvelle direction à ma vie* | - | 0.80 | - | - |
| 11. I’m able to do better things with my life  *Je fais de ma vie quelque chose de meilleur* | - | 0.82 | - | - |
| 14. New opportunities are available which wouldn’t have been otherwise  *De nouvelles opportunités sont apparues* | - | 0.65 | - | - |
| 17. I’m more likely to try to change things which need changing  *J’essaie davantage de changer les choses qui ont besoin d'être changées* | - | 0.71 | - | - |
| 1. I changed my priorities about what is important in life  *J’ai changé de priorités dans la vie* | - | 0.63 | - | - |
| ***Personal capacities (PC)*** |  |  |  |  |
| 4. I have a greater feeling of self-reliance  *J’ai acquis plus confiance en moi* | - | - | 0.73 | - |
| 10. I know better that I can handle difficulties  *Je suis davantage capable de gérer des situations difficiles* | - | - | 0.75 | - |
| 12. I am better able to accept the way things work out  *J’accepte mieux la façon dont les choses se passent* | - | - | 0.78 | - |
| 19. I discovered that I’m stronger than I thought I was  *J’ai découvert que je suis plus fort(e) que ce que je pensais* | - | - | 0.65 | - |
| 2. I have a greater appreciation for the value of my own life  *J’apprécie plus ma vie à sa vraie valeur* | - | - | 0.75 | - |
| 13. I can better appreciate each day  *J’apprécie plus amplement chaque jour de ma vie* | - | - | 0.80 | - |
| ***Spiritual change (SC)*** |  |  |  |  |
| 5. I have a better understanding of spiritual matters  *J’ai développé une certaine spiritualité* | - | - | - | 0.93 |
| 18. I have a stronger religious faith  *J’ai une foi religieuse plus grande* | - | - | - | 0.83 |

**Table S2**: Standardized factor loadings of the 21 items of the posttraumatic growth inventory obtained after the oblique confirmatory factor analysis based on four-factor structure evidenced by the Clustering around Latent Variable analysis


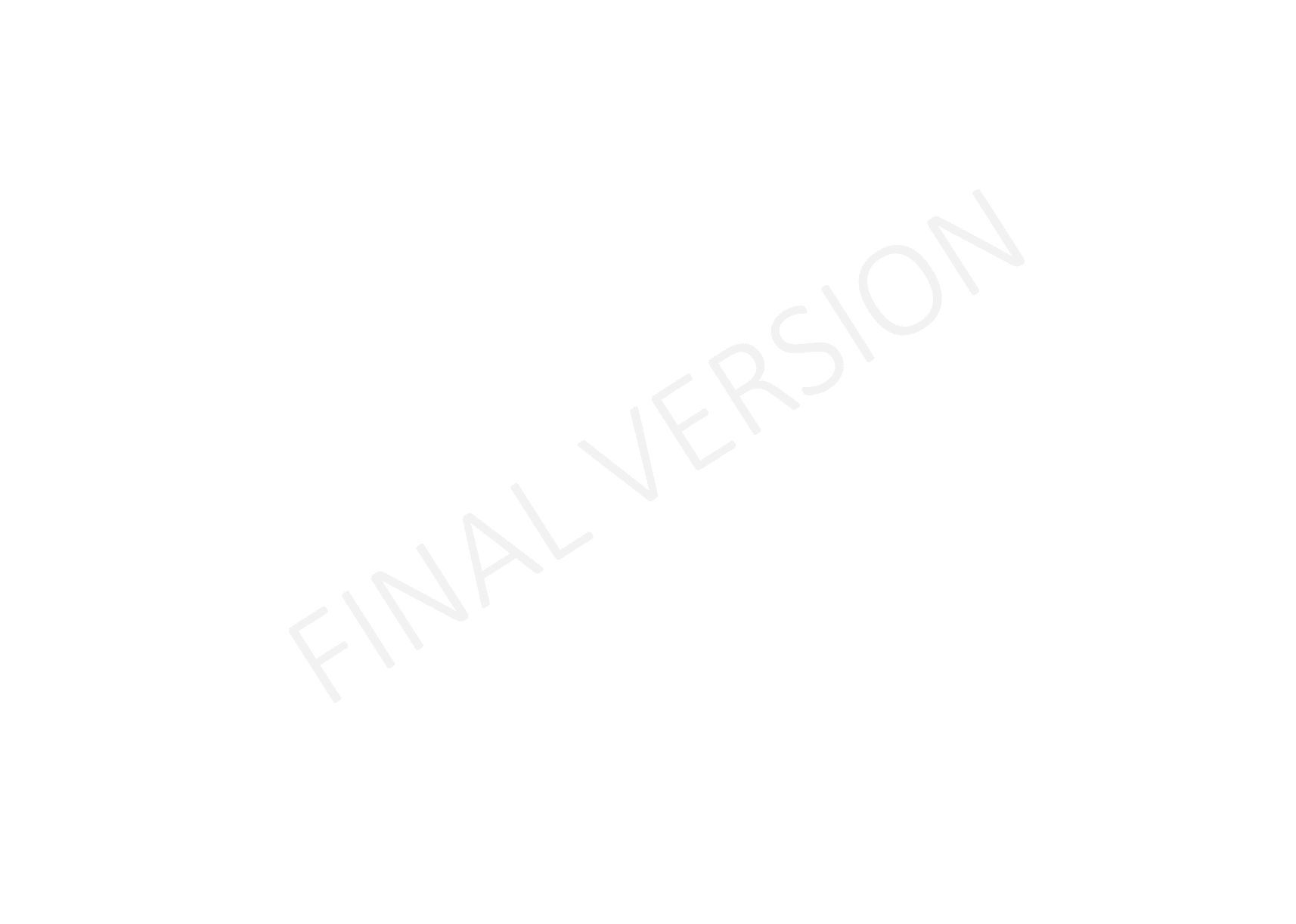
**Appendix D:** The final French version of the posttraumatic growth inventory that we propose, with five response categories per item and four domains (resulting from the analyses of the manuscript)

**Inventaire du Développement Post-Traumatique**

| Du fait de mon cancer : | Pas du tout  0 | Très peu  1 | Un peu  2 | Beaucoup  3 | Totalement  4 |
| --- | --- | --- | --- | --- | --- |
| 1. J’ai changé de priorités dans la vie (NLD) |  |  |  |  |  |
| 2. J’apprécie plus ma vie à sa vraie valeur (PC) |  |  |  |  |  |
| 3. Je me suis intéressé(e) à de nouvelles choses (NLD) |  |  |  |  |  |
| 4. J’ai acquis plus confiance en moi (PC) |  |  |  |  |  |
| 5. J’ai développé une certaine spiritualité (SC) |  |  |  |  |  |
| 6. Je vois mieux que je peux compter sur les autres en cas de problème (RO) |  |  |  |  |  |
| 7. J’ai donné une nouvelle direction à ma vie (NLD) |  |  |  |  |  |
| 8. Je me sens plus proche des autres (RO) |  |  |  |  |  |
| 9. Je suis plus enclin(e) à exprimer mes émotions (RO) |  |  |  |  |  |
| 10. Je suis davantage capable de gérer des situations difficiles (PC) |  |  |  |  |  |
| 11. Je fais de ma vie quelque chose de meilleur (NLD) |  |  |  |  |  |
| 12. J’accepte mieux la façon dont les choses se passent (PC) |  |  |  |  |  |
| 13. J’apprécie plus amplement chaque jour de ma vie (PC) |  |  |  |  |  |
| 14. De nouvelles opportunités sont apparues (NLD) |  |  |  |  |  |
| 15. J’ai plus de compassion pour les autres (RO) |  |  |  |  |  |
| 16. J’investis plus mes relations aux autres (RO) |  |  |  |  |  |
| 17. J’essaie davantage de changer les choses qui ont besoin d'être changées (NLD) |  |  |  |  |  |
| 18. J’ai une foi religieuse plus grande (SC) |  |  |  |  |  |
| 19. J’ai découvert que je suis plus fort(e) que ce que je pensais (PC) |  |  |  |  |  |
| 20. Je vois plus le bon côté des gens (RO) |  |  |  |  |  |
| 21. J’accepte mieux le fait d'avoir besoin des autres (RO) |  |  |  |  |  |
| *Notes. Items are highlighted according to their domain (domains are also shown for each item in brackets): NLD: New life direction / Nouvelle direction pour la vie (6 items), PC: Personal capacities / Capacités personelles (6 items), SC: Spiritual Chance / Changement spirituel (2 items), RO: Relating to others / Relation aux autres (7 items)* | | | | | |
